# ‘Medusa head ataxia’: the expanding spectrum of Purkinje cell antibodies in autoimmune cerebellar ataxia. Part 1: Anti-mGluR1, anti-Homer-3, anti-Sj/ITPR1 and anti-CARP VIII

**DOI:** 10.1186/s12974-015-0356-y

**Published:** 2015-09-17

**Authors:** S. Jarius, B. Wildemann

**Affiliations:** Molecular Neuroimmunology Group, Department of Neurology, University of Heidelberg, Otto Meyerhof Center, Im Neuenheimer Feld 350, D-69120 Heidelberg, Germany

**Keywords:** Autoimmune cerebellar ataxia, Cerebellitis, Paraneoplastic cerebellar degeneration, Autoantibodies, Purkinje cells, Metabotropic glutamate receptor 1 (mGluR1) antibodies, Homer-3 antibodies, Anti-Sj, Inositol 1,4,5-trisphosphate receptor 1 (ITPR1, I3PR) antibodies, Carbonic anhydrase-related protein VIII (CARP VIII) antibodies, Protein kinase gamma (PKCγ) antibodies, Anti-Ca, Rho GTPase-activating protein 26 (ARHGAP26, GRAF) antibodies, Glutamate receptor delta2 (GluRδ2) antibodies, Anti-Yo, Cerebellar degeneration-related protein 2 (CDR2) antibodies, Cerebellar degeneration-related protein 2-like (CDR2L) antibodies, Purkinje cell antibody 2 (PCA-2), Anti-Tr, Delta notch-like epidermal growth factor-related receptor (DNER) antibodies, Anti-Nb, Anti-AP3B2, Neuronal adaptin-like protein (beta-NAP) antibodies, Voltage-gated calcium channel (VGCC) antibodies

## Abstract

Serological testing for anti-neural autoantibodies is important in patients presenting with idiopathic cerebellar ataxia, since these autoantibodies may indicate cancer, determine treatment and predict prognosis. While some of them target nuclear antigens present in all or most CNS neurons (e.g. anti-Hu, anti-Ri), others more specifically target antigens present in the cytoplasm or plasma membrane of Purkinje cells (PC). In this series of articles, we provide a detailed review of the clinical and paraclinical features, oncological, therapeutic and prognostic implications, pathogenetic relevance, and differential laboratory diagnosis of the 12 most common PC autoantibodies (often referred to as ‘Medusa-head antibodies’ due to their characteristic somatodendritic binding pattern when tested by immunohistochemistry). To assist immunologists and neurologists in diagnosing these disorders, typical high-resolution immunohistochemical images of all 12 reactivities are presented, diagnostic pitfalls discussed and all currently available assays reviewed. Of note, most of these antibodies target antigens involved in the mGluR1/calcium pathway essential for PC function and survival. Many of the antigens also play a role in spinocerebellar ataxia. Part 1 focuses on anti-metabotropic glutamate receptor 1-, anti-Homer protein homolog 3-, anti-Sj/inositol 1,4,5-trisphosphate receptor- and anti-carbonic anhydrase-related protein VIII-associated autoimmune cerebellar ataxia (ACA); part 2 covers anti-protein kinase C gamma-, anti-glutamate receptor delta-2-, anti-Ca/RhoGTPase-activating protein 26- and anti-voltage-gated calcium channel-associated ACA; and part 3 reviews the current knowledge on anti-Tr/delta notch-like epidermal growth factor-related receptor-, anti-Nb/AP3B2-, anti-Yo/cerebellar degeneration-related protein 2- and Purkinje cell antibody 2-associated ACA, discusses differential diagnostic aspects and provides a summary and outlook.

## Introduction

Autoimmune cerebellar ataxia (ACA) is an important differential diagnosis in patients presenting with signs and symptoms of cerebellar disease. Alongside multiple sclerosis and acute disseminated encephalomyelitis, autoantibody-associated disorders of the CNS are the most common cause of ACA. While ACA is a rare manifestation in some of these disorders, e.g. aquaporin-4 (AQP4) antibody-associated neuromyelitis optica (NMO), it is the most frequent or exclusive presentation in others. To date, around 30 different autoantibodies targeting brain antigens have been reported in patients with ACA, many of which are of paraneoplastic origin (Table 1).

**Table 1:** Selected antibodies to cerebellar antigens reported in patients with cerebellar ataxia

Target structures	Comments	Ref
*Purkinje cells*		
*MGluR1/calcium pathway-related*		
Anti-mGluR1	Tumour-associated in some cases	[33–36]
Anti-Homer-3	Lung cancer-associated in one unpublished cases	[85, 86]
Anti-Sj/ITPR1	NSCLC-associated in one unpublished case	[100]
Anti-CARP VIII	Reported in association with melanoma and ovarian cancer	[134, 135]
Anti-PKCγ	Reported in association with SCLC and liver cancer	[147, 148]
Anti-GluRδ2	Mostly para/postinfectious	[149–151]
Anti-Ca/ARHGAP26	Tumour-associated in a few cases	[74, 152, 153]
Anti-P/Q-type VGCC	Tumour-associated in many cases	[13, 14]
Anti-N type VGCC	Often associated with anti-P/Q-type VGCC	[154–157]
Anti-Yo/CDR2 (PCA-1)^a^	Typical paraneoplastic syndrome	[15, 158–162]
Anti-Nb/AP3B2/beta-NAP	Tumour-association unknown	[163, 164]
*Others*		
PCA-2 (target antigen not known)	Tumour-associated in almost all published cases	[154]
Anti-Tr/DNER	HD-associated in almost all cases	[165–169]
*Molecular and granular layer, PCs spared*		
Anti-amphiphysin	Tumour-associated in most cases	[170]
Anti-GABABR	Tumour-associated in many cases	[50–52]
Anti-DPPX	Reported in association with B cell neoplasm in a few patients	[171–174]
Anti-Caspr2	Facultatively paraneoplastic	[175, 176]
*Pinceau formation/Basket cells*		
Anti-LGI1	Mainly not tumour-associated	[177]
*Granular layer*		
Anti-GAD	DM-associated (mostly DM type I) and, in neurological patients, often tumour-associated	[119, 178–181]
*Oligodendrocytes*		
Anti-CV2/CRMP5	Typical paraneoplastic syndrome	[182–184]
Anti-MOG	Usually non-paraneoplastic	[185, 186]
*Astrocytic endfeet*		
Anti-AQP4	Very rarely causing cerebellar ataxia, usually non-paraneoplastic	[187, 188]
*Neuronal nuclei*		
ANNA-1 (Anti-Hu/HuD)	Neuronal nuclei in the CNS and PNS paraneoplastic	[189–191]
ANNA-2 (Anti-Ri)	Neuronal nuclei in the CNS paraneoplastic	[192, 193]
ANNA-3 (unknown antigen)	Typical paraneoplastic syndrome	[194]
Anti-Zic4	Typical paraneoplastic syndrome	[195, 196]
Anti-Zic2	Mostly SCLC-associated	[197]
Anti-Zic1	Mostly SCLC-associated	[197]
*Bergman glial cell nuclei*		
AGNA/Anti-SOX1^b^	Typically tumour-associated	[198, 199]
*Nucleoli*		
Anti-Ma2/Ta (PNMA2)	Typical paraneoplastic syndrome	[200, 201]
Anti-Ma1 (PNMA1)	Typical paraneoplastic syndrome	[200, 201]
*Centrosome*		
Anti-γγ-enolase, -pericentrin, -ninein, -PCM1, -Mob1	Para-/post varicella zoster virus	[202]
*Centriols*		
Anti-centriolar antibodies	Para-/post M. pneumoniae	[203]
*Others*		
Anti-transglutaminase6	Associated with celiac disease	[204, 205]
Anti-triophosphate isomerase	Post-EBV	[206]
Anti-20 S proteasome	Associated with anti-Yo	[207]
Anti-GQ1b	‘Ataxic Guillaine Barré syndrome’	[208–211]

*DM* diabetes mellitus, *HD* Hodgkin’s disease

^a^Further target antigens reported in the literature: CDR34, CDR3, CDR2L

^b^Whether AGNA and SOX1 are identical is controversial; recent evidence suggests that they may represent different reactivities

**Fig. 1:**
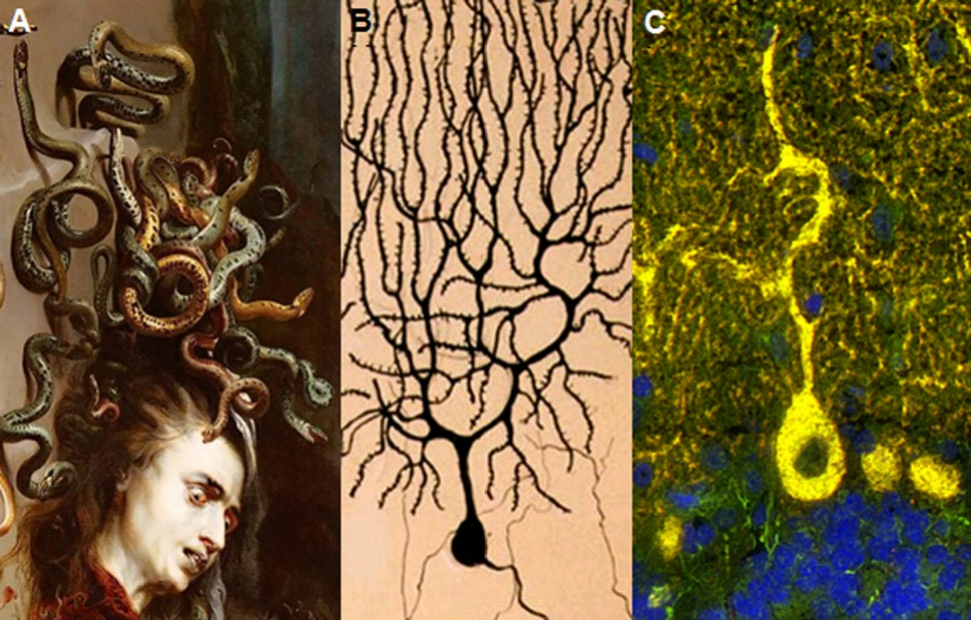
Medusa-head ataxia. **a** Detail from Sir Peter Paul Ruben’s (1577–1640) famous painting of a gorgon head (dated 1617/1618; Kunsthistorisches Museum, Vienna, Austria). **b** A drawing of a Purkinje cell by the Spanish pathologist, histologist, neuroscientist, and Nobel laureate Santiago Felipe Ramón y Cajal (1852–1934). **c** Purkinje cells somata and dendrites stained by IgG from a patient with autoimmune cerebellar ataxia

When tested by immunohistochemistry (IHC) using cerebellum tissue sections, some of these antibodies (anti-metabotropic glutamate receptor 1 (mGluR1), anti-Homer protein homolog 3 (Homer-3), anti-Sj/inositol 1,4,5-trisphosphate receptor (ITPR1), anti-carbonic anhydrase-related protein VIII (CARP VIII), anti-protein kinase C gamma (PKCγ), anti-Ca/RhoGTPase-activating protein 26 (ARHGAP26), anti-glutamate receptor delta 2 (GluRδ2), anti-Tr/delta notch-like epidermal growth factor (EGF)-related receptor (DNER), voltage-gated calcium channels (VGCC) antibodies, anti-Nb/AP3B2, anti-Yo/cerebellar degeneration-related protein 2 (CDR2) and Purkinje cell antibody 2 (PCA-2)) show a staining pattern resembling a Gorgon’s head, caused by binding of IgG to Purkinje cell (PC) somata and dendrites and are therefore often referred to as ‘Medusa head’ antibodies (Fig. 1).

Due to their similar binding patterns, it can be very difficult to differentiate the members of the expanding family of somatodendritic or ‘Medusa head’ PC antibodies. Here, we show exemplary IHC findings for each of these antibodies, review the currently available diagnostic assays, and discuss diagnostic pitfalls. In addition, we provide a comprehensive summary of the clinical, paraclinical and epidemiological features associated with these antibodies, briefly review the available knowledge regarding their pathophysiological relevance and discuss their oncological and prognostic implications.

The present, first article in this series will review the current knowledge on anti-mGluR1-, anti-Homer-3-, anti-Sj/ITPR1- and anti-CARP VIII-positive ACA.

## Antibodies targeting antigens involved in the glutamate/calcium pathway

Interestingly, most of the antigens so far identified in patients with ‘Medusa head’ antibodies—namely mGluR1, Homer-3, ITPR1, CARP VIII, PKCγ, GluRδ2, VGCC, CDR2-like (CDR2L), neuronal adaptin-like protein (beta-NAP) and possibly also ARHGAP26 and CDR2—are functionally and structurally related in that all are involved in the PC phosphatidylinositol-calcium second messenger system or, more generally, in maintaining intracellular calcium homeostasis: In the cerebellum, release of glutamate, the major excitatory neurotransmitter in the CNS, by parallel fibre (PF) (and possibly climbing fibre (CF)) [1] synapses stimulates postsynaptic mGluR1, which is the main metabotropic receptor on PCs. At the PC/PF synapse, this results in cleavage of phosphatidyl 4,5-bisphosphate (PIP_2_) by phospholipase Cβ3 (PLCβ3), the target of the G proteins of mGluR1, into diacylglycerol (DAG) and inositol 1,4,5-trisphosphate (IP3). IP3 acts as a second messenger for ITPR1, a calcium channel mainly located in the membrane of the smooth endoplasmic reticulum (ER) and physically linked to mGluR1 via Homer-3 [1]. Upon activation by IP3, ITPR1 mediates intracellular Ca^2+^ release from the ER calcium storage [1]. CARP VIII, on the other hand, limits Ca2+ efflux from the ER by reducing the affinity of ITPR1 for IP3 [2]. Intracellular calcium together with DAG activates PKCγ [3], a protein kinase involved in calcium regulation by its capability to phosphorylate and thus inactivate the DAG-activated canonical transient receptor potential (TRPC) type 3 cation channel 3 [4–6], resulting in reduced influx of calcium ions [7]. GluRδ2 has been recently shown to associate with mGluR1, PKCγ and TRPC3 [5, 6, 8–10] and to regulate mGluR1-mediated synaptic transmission in PCs. ARHGAP26 has been found to precipitate with dynamin, which is involved in mGluR1 internalisation [11, 12]. Blockade of CDR2 by anti-Yo autoantibodies has been reported to induce the expression of PKCγ and the pore- and gating apparatus-forming VGCC protein Ca_v_2.1, which is an autoantigen in ACA itself [13, 14], and also to regulate the expression of several other calcium-related proteins [15]. Finally, mGluR1a interacts directly with Ca_v_2.1, forming a heteromeric protein complex [16–18] and has been shown to inhibit Ca_v_2.1-mediated Ca2+ currents [16, 19, 20].

Of note, mutations in almost all of the components of the mGluR1 cascade have been demonstrated to cause cerebellar ataxia either in humans or in animal models. Homozygous mutations in the GRM1 gene encoding mGluR1 underlie spinocerebellar ataxia (SCA) 13 [21]. G_q_ mutant PCs remain multiply innervated by CFs and are associated with impaired motor coordination in adult mice [22, 23]. Phospholipase C mutant mice show deficient long-term synaptic depression and impaired motor learning [24]. Mutations in the ITPR1 gene have been found to cause SCA15 and 21 [25]. Missense mutations in the PKCγ gene have been found in SCA14 [26], and loss of PKCγ also seems to play a role in SCA1 [27]. Mice deficient in TRPC3 exhibit impaired walking behaviour [5]. Mutations in beta-3 spectrin influencing glutamate receptor GluRδ2 expression as well as deletions in the GRID2 gene itself have recently been discovered in SCA5 and other forms of hereditary cerebellar ataxia in humans [28, 29]. Finally, mutations in the Ca_v_2.1 gene cause SCA6 [30–32].

All sections dealing with individual antibody reactivities are structured uniformly to improve accessibility of the information provided. Each section consists of an identically headed set of subsections dealing with (1) clinical, paraclinical and epidemiological features associated with the respective antibody; (2) associated tumours; (3) syndrome outcome and prognosis; (4) target antigen structure and function; (5) diagnostic IHC findings; (6) antigen-specific assays; (7) relevance of CSF testing; (8) association with other autoantibodies; (9) evidence for a pathogenic role of the antibody; and (10) molecular genetics, inasmuch as they corroborate a potential role of the target antigen in cerebellar ataxia.

## Anti-mGluR1

### Clinical, paraclinical and epidemiological features

Since the first description of anti-mGluR1 in 2000, five patients have been reported (three female, two male; median age 50 years, range 19–69) [33–36], all of whom presented with cerebellar gait ataxia (partly unable to walk without help) and limb ataxia (dysmetria of arms and/or legs, intention tremor). Further symptoms included trunk ataxia (partly unable to sit without help, head titubation, truncal sway) in four, dysarthria in four, and ocular symptoms (nystagmus, oscillopsia, diminution in speed of saccades, impaired adaptation of saccadic eye movements, difficulty directing and maintaining fixation of gaze, slight opsoclonus) in all cases. While a subacute onset was noted in two, symptoms worsened slowly in two other patients (no data in one). Magnetic resonance imaging (MRI) showed cerebellar atrophy in two patients [34, 36] and diffuse abnormal hyperintensity in the whole cerebellum present only on fluid-attenuated inversion recovery and diffusion sequences in another case [35], but was normal in the remaining two (follow-up for up to 6 months). Lumbar puncture revealed mononuclear pleocytosis in three patients (9, 28, and 190 cells/μl) and was normal in one (no data in one case); signs of intrathecal IgG synthesis were present in one of two patients examined. Additional cases of ACA and mGluR1 have been identified at the authors’ institution and elsewhere, but no additional clinical information is currently available.

Given that mGluR1 is expressed widely throughout the CNS, it is not surprising that two patients developed signs of encephalitis in addition to ataxia, including mild cognitive decline in one case and short-term memory loss in the other.

#### Association with tumours

In three out of five cases, anti-mGluR1 autoantibodies were associated with malignant tumours. The two index patients had a history of nodular sclerosing Hodgkin’s disease (HD) but had been in remission for 2 and 9 years, respectively, at the time of onset of anti-mGluR1-associated ACA; however, mGluR1 was not detected in a tumoral lymph node from one of those patients, and no tumour specimen was analysed in the second case, rendering it unclear whether the two conditions were pathophysiologically related [33]. Sera from patients with Hodgkin’s lymphoma but no cerebellar ataxia did not show anti-mGluR1 [33]. A third patient had an adenocarcinoma of the prostate, which was only discovered 20 months after onset of the cerebellar ataxia, as well as a history of a successfully treated cutaneous T cell lymphoma. In contrast to the index cases, mGluR1 was found to be abundantly present in the tumour tissue and binding of the patient’s IgG to tumoral mGluR1 could be demonstrated [34]. Two patients did not show any evidence of a tumour up to 40 months after onset [35, 36].

#### Outcome and prognosis

While treatment with steroids, plasma exchange (PEX), intravenous immunoglobulins (IVIG) and oral steroids was followed by slow yet complete recovery in index patient 1; PEX did not result in significant improvement in the second patient, who remained unable to walk without support. Hodgkin’s disease remained in complete remission in both cases [33]. In a third patient, commencement of treatment with steroids, IVIG and mycophenolate mofetil early in the disease course led to continuous clinical improvement and a drop in anti-mGluR1 serum titres (1:20,000 to 1:500). At last follow-up, 40 months after onset, the patient was still able to walk [35]. In patient 4, a transient improvement was noted after intravenous methylprednisolone (IVMP); however, subsequent courses of IVMP were not followed by further improvement, and severe and disabling ataxia and dysarthria were present at last follow-up [36]. In patient 5, treatment of the prostate carcinoma was associated with severe neurological deterioration; later on, sustained improvement was achieved after treatment with IVIG and low-dose steroids [34].

#### Antigen

MGluR1 (encoded by GRM1) is a cell surface receptor belonging to the guanine nucleotide-binding protein (G-protein)-activating receptor 3 family [37]. Its natural ligand is the excitatory neurotransmitter L-glutamate. Glutamate produces fast excitation through activation of ionotropic glutamate receptors (GluRs, including *N*-methyl *D*-aspartate (NMDA) receptors, α-amino-3-hydroxy-5-methyl-4-isoxazolepropionic acid (AMPA) receptors and kainate (KA) receptors) and slower actions through metabotropic receptors (mGluRs). To date, eight mGluRs are known (mGluR1-8). Together with mGluR5, mGluR1 forms group I of the metabotropic glutamate receptors. So far, five isoforms of mGluR1 have been described [37–39] with the canonical isoform alpha being a disulphide-linked homodimer primarily coupled to G_q_/G_11_ [40] by which it is linked to the inositol phospholipid metabolism, i.e. it elicits an increase in the PIP_2_ turnover by activating PLCβ to hydrolyse PIP_2_ to IP3 and DAG, which results in intracellular calcium release from intracellular stores and activation of PKCγ. Besides classical, glutamate-stimulated activation, also agonist-independent, ‘constitutive’ activity of mGluR1 (and mGluR5) occurs, modulated by intracellular proteins including Homer-3 and Homer-1a [41].

The protein comprises an extracellular N-terminus containing the glutamate binding site, seven alpha-helical transmembrane domains and an (isoform-specific) cytoplasmic C-terminus—with the exception of isoform 1e, which is truncated before the first transmembrane domain. The G-protein-binding C-terminus contains domains that regulate mGluR1 function/signalling as well as its localisation and subcellular distribution in the dendritic membrane, its trafficking and its internalisation. Importantly, via a cytoplasmic Homer-binding PPxxFR motif, the receptor (more specifically, the long 1194-amino acid isoform 1a [42, 43]) binds Homer-3 (as well as Homer-1 and -2), another autoantigen in ACA, which regulates the postsynaptic localisation of mGluR1 as well as its activity [44].

In common with other mGluRs, the postsynaptic group I mGluRs transduce stimulatory signals at excitatory synapses. MGluR1 is present in the highest concentrations at the PF/PC synapse. Upon stimulation, the receptor modulates neuronal excitability by controlling ion channels. Modifications in the subcellular expression and distribution of mGluR1, together with changes induced by stimulation of mGluR1, participate in the long-term synaptic plasticity involved in memory formation and learning [37]. MGluR1 regulation is believed to have an important role in both types of long-term synaptic plasticity: while it has been implicated in long-term depression (LTD) of synaptic efficacy in the cerebellum, it is involved in long-term potentiation (LTP) in the hippocampus [45–47].

The proteins with which mGluR1 is associated or interacts in the cerebellum, hippocampus or cerebral cortex include, among others, TRPC, a cation channel involved in slow excitatory cation conductance [5, 48], the P/Q-type voltage-gated calcium channel (VGCC) (Ca_v_2.1) [16] and gamma-aminobutyric acid type B receptors [49], which are both known target autoantigens in patients with ACA themselves [13, 50–52], NMDA receptors [53, 54], an important autoantigen in autoimmune encephalitis [55], and adenosine A1 receptor [56].

Outside the cerebellum, mGluR1 has been found in mitral and tufted cells of the olfactory bulb [57] and, at lower levels, in the hippocampus [58], the amygdala, the hypothalamus, where they take part in regulating circadian rhythms [59] and hormone secretion [60], the basal ganglia including the subthalamic nucleus, the thalamus, where it is involved in processing of nociception and pain and of other sensory information [61–64], and the ventral horn, central grey, substantia gelatinosa and sensory trigeminal nuclei of the spinal cord, where it is also implicated in nociception, as well as in the cerebral cortex and brainstem [38, 65, 66]; mGluR1 has important physiological roles also in motor neurons [67–69].

#### Immunohistochemistry

**Fig. 2:**
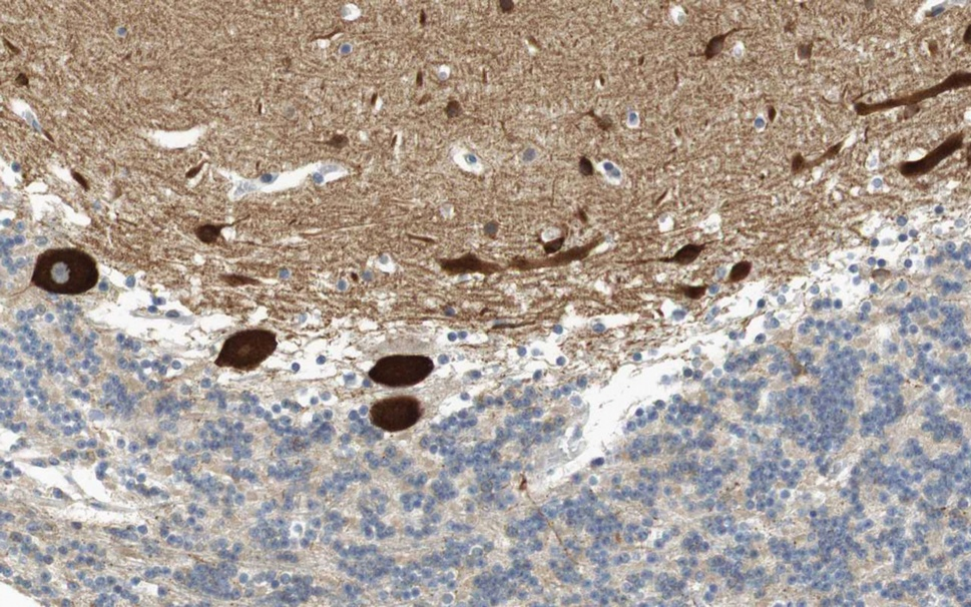
Expression of mGluR1 in the human cerebellum as demonstrated by IHC (modified image from the Human Protein Atlas image database [101])

As indicated above, mGluR1 is widely expressed throughout the central nervous system, mostly postsynaptically in neuronal dendrites and somata [38, 70, 71]. Within the cerebellum, it is enriched in PCs, with the highest levels in the dendritic spines (Fig. 2) [38, 58, 65]; in addition, granular cells and other interneurons seem to express mGluR1 at lower RNA and protein levels [58, 66, 72, 73]. Makoff et al. [39] found mGluR1c exclusively in granule cells by in situ hybridisation, while an mGLUR1a/mGLUR1b probe reacted in addition with PCs and basket cells. Mateos et al. [71] found both mGluR1a and mGluR1b by immunogold labelling in the dendritic spines of PCs receiving PF synaptic terminals and reported additional peri-extrasynaptic mGluR1a/b expression.

**Fig. 3:**
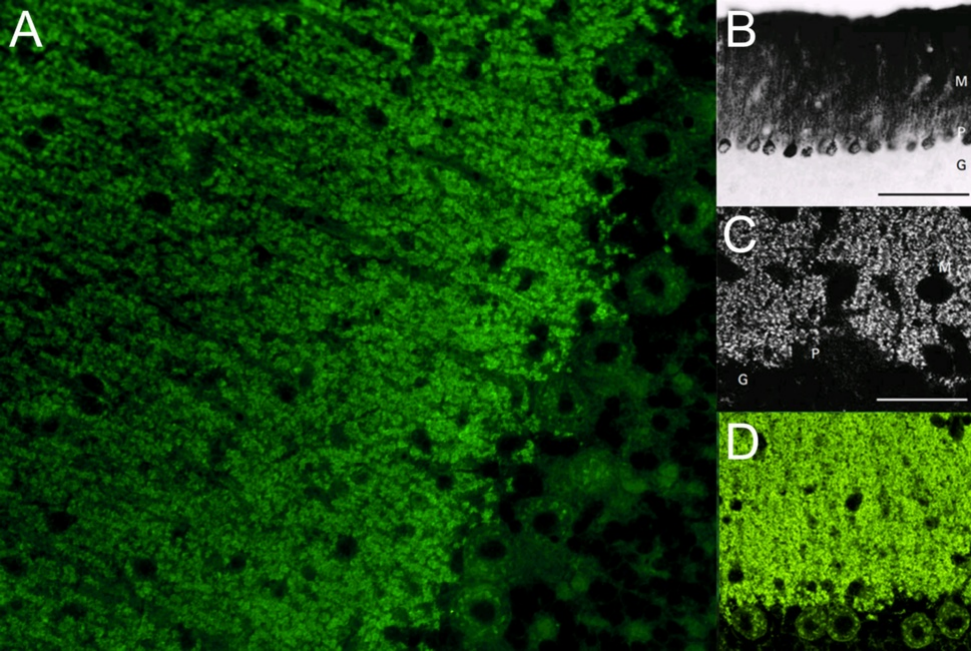
Binding of serum anti-mGluR1 from patients with ACA to rat (panels **a** and **d**) and mouse (panels **b** and **c**) cerebellum tissue sections. Patient antibodies were detected by use of goat anti-human IgG secondary antibodies labelled with Alexa Fluor®488 (panel **a** and **d**) or biotin (panel **b**: avidin/peroxidase; panel **c**: avidin/Texas red). While anti-mGluR1 strongly stain the molecular layer (**a**-**d**), staining of the PC somata varies in intensity depending on detection methods and antibody titres (compare panels **a** and **c** to panels **b** and **d**). Permission for panels **b** and **c** obtained from Massachusetts Medical Society. Copyright © (2000) Massachusetts Medical Society. Sillevis Smitt et al., Paraneoplastic cerebellar ataxia due to autoantibodies against a glutamate receptor. N Engl J Med. 2000; 342:21-27

Anti-mGluR1 autoantibodies were originally detected by avidin-biotin peroxidase and avidin Texas red IHC, respectively, using formalin-perfused sections of mouse and human cerebellum [33]. The patients’ sera strongly stained PC bodies and dendrites (but not the PC axons). Using confocal microscopy, a strong punctate staining in the molecular layer of the cerebellum was observed, indicative of labelling of the PC spines. Later studies used mouse, rat or primate brain sections (formalin fixed in three studies, not specified in another one) and either conventional IHC or indirect immunofluorescence (IIF) and reported a similar binding pattern [34–36, 74]. The punctate staining seen with anti-mGluR1-positive sera was considered different from that reported for anti-Tr [35]. Outside the cerebellum, strong staining of neurons and neuropil was observed in the glomeruli of the olfactory bulb, the olfactory tubercle (including the islands of Calleja), the superficial layer of the cerebral cortex, the thalamus, the superior colliculus, the spinal trigeminal nucleus and the CA3 area [33] and dentate gyrus [34] of the hippocampus. See Fig. 3 for typical IHC findings.

#### Antigen-specific assays

A cell-based assay (CBA) employing human embryonic kidney 293 (HEK293) cells transfected with human mGluR1a (Euroimmun, Luebeck, Germany) is available at the authors’ institution for use in scientific studies. Several other HEK293 or Chinese hamster ovary (CHO) in-house CBAs employing rat, mouse or human mGluR1 have been reported [33–36]. The patient antibodies were shown to bind to isoform a in at least two studies (not specified in the remaining studies) [33, 34]. Other approaches to demonstrate specificity for mGluR1 included the use of cerebellum sections obtained from mGluR1-knockout mice, resulting in abolition of the typical staining pattern [33, 35], and an mGluR1 inhibition assay based on measurement of the glutamate-stimulated formation of inositol phosphates in CHO-transfected cells before and after incubation with patient serum [33].

#### CSF testing

The two index patients were positive for mGluR1 both in serum and in the CSF. However, titres per unit of IgG were 31 and 36 times as high in the CSF as in serum, indicating intrathecal synthesis [33]. In a third patient, a serum titre of 1:20,000 and a CSF titre of 1:500 were found in a fluorescence-based IHC assay [35]. CSF samples also tested positive in four studies of mGluR1-specific CBAs [33–36].

#### Association with other autoantibodies

No association of anti-mGluR1 antibodies with other anti-neural antibodies (including anti-Hu, -Yo, -Ri, -Tr, -CV2/CRMP5, -Ma/Ta, -glutamic acid decarboxylase (GAD), -NMDAR, -AMPAR, -GABABR, -glycine receptors, leucine-rich, glioma inactivated 1 (LGI1), contactin-associated protein-2 (CASPR2), -amphiphysin, -Homer-3 and –Tr/DNER) has been found so far [33–36]. Cross-reactivity with the structurally closely related mGluR5 receptor was excluded in three patients using HEK293- or CHO-based CBAs [33, 36].

#### Pathogenetic relevance

As a plasma membrane protein with a large extracellular domain [38, 39], mGluR1 is accessible to circulating IgG. Three independent studies consistently showed that anti-mGluR1 indeed targets the N-terminal, ligand-binding extracellular domain of native mGluR1a as indicated by their binding to living, i.e. unfixed, CHO or HEK cells expressing mGluR1 and by the lack of effect of anti-mGluR1 on PC function if injected intracellularly instead of being added extracellularly [33, 34, 75].

In contrast to most other antibodies discussed here, passive transfer experiments have been performed and strongly indicated a direct pathogenic effect of the antibody. Transfer of anti-mGluR1 into the subarachnoid space of normal mice, near the cerebellum, causes increasing ataxia with a wide and uncoordinated, irregular gait and a pathological rotorod test. The most strongly affected mice could hardly stand up owing to severe truncal ataxia [33]. Post-mortem analysis showed IgG deposits mainly in the cerebellum, including the cerebellar cortex [33]. The fact that antibodies eluted from mGluR1a-expressing CHO cells incubated with patient serum caused similar ataxic symptoms, while sera preadsorbed with such cells did not, proves that the effects were elicited by anti-mGluR1 and not by other antibodies potentially present in the patient serum [33].

As no freshly frozen human serum was co-injected as complement source and as the effect set in after a very short time and spontaneously subsided after 24 h, it is likely that ataxia was caused by functional blocking of mGluR1. Evidence for a functional impact of anti-mGluR1 on the receptor also comes from the demonstration of a dose-dependent decrease in glutamate-induced inositol phosphate formation following incubation of mGluR1a-expressing CHO cells with patient (but not control) IgG [33]. Similarly, IgG from anti-mGluR1-positive serum acutely reduced the holding inward current of PC in a slice culture model and markedly suppressed the inward current induced by (RS)-3,5-dihydroxyphenylglycine (DHPG), a selective agonist for group I mGluRs. The latter effect was reversible by a 20- to 40-min wash [75]. When incubated with spontaneously (unstimulated) firing PCs, a slight hyperpolarisation and thus hypoexcitability and a significant reduction in the action potential firing rate was noted [75]. Moreover, when applied in vivo directly to the flocculus of mice by a minipump, anti-mGluR1 (but not control IgG) strongly and acutely disturbed the visual component of compensatory eye movements as indicated by a reduction in the amplitude of the optokinetic reflex as well as the vestibulo–ocular reflex response in light; the effect was reversible by removal of the pump [75]. Finally, application of mGluR1 to cultured embryonic mouse PC during LTD induction strongly attenuated the LTD-defining decrease in the amplitude of the excitatory postsynaptic current following glutamate/depolarisation conjunctive stimulation at the PF/PC synapse [75]. While the calcium influx was unaltered, calcium mobilisation was significantly reduced, in line with the reduction in mGluR1-mediated inward current and the reduction of phosphatidylinositol turnover measured in mGluR1-expressing CHO cells [75].

Whether complement- or cell-mediated, antibody-related cytotoxicity is involved in the pathogenesis, in vivo has not been investigated thus far. Considering the prominent role of group I mGluRs in neuroprotection, blockade of the receptor might result in PC cell loss also in the absence of a strong immune reaction [76–78]. Coesmans et al. [75] indeed found an (up to two third) decrease in the density of PCs in all parts of the cerebellar hemispheres and vermis in a post-mortem analysis of a patient, who had died from cardiac infarction. Of note, no signs of an ongoing inflammation (including cytotoxic CD8+ T lymphocytes previously reported in other types of ACA) were noted despite severe persisting ataxia at the time of death. In areas with PC loss, reactive Bergmann gliosis was present [75]. Moreover, PC morphology was affected with the dendritic trees of the remaining PCs being severely amputated [75]. In accordance with that finding, cerebellar atrophy indirectly indicating cell loss was detected by MRI in two further patients [34, 36].

Indirect evidence for a pathogenic role of the antibody comes from the demonstration of mGluR1-specific plasma cell clones within the CNS [33, 35] and from the fact that fading of ataxia after immunotherapy was paralleled by disappearance of the antibody [33]; by contrast, persistence of ataxia was accompanied by persisting serum and CSF anti-mGluR1 in a second patient [33].

Considering that only a subset of patients with mGluR1 reported so far had an accompanying tumour and that the receptor was not detectable in tumour samples renders a simple paraneoplastic aetiology caused to ectopic protein expression unlikely.

#### Molecular genetics

A pathogenic impact of anti-mGluR1 is also supported by molecular genetic findings linking mGluR1 dysfunction to cerebellar ataxia. Most importantly, autosomal recessive spinocerebellar ataxia-13 (SCA13) has been found to be caused by a complex homozygous mutation in the GRM1 gene encoding mGluR1 that results in aberrant transcripts lacking important functional domains [21]. SCA13 is a slowly progressive CNS disorder with onset in infancy that is characterised by moderate to severe gait, stance and limb ataxia with dysmetria, tremor, dysdiadochokinesia and dysarthria, and generalised cerebellar atrophy on MRI with small inferior vermis and retrocerebellar cysts, eye movement abnormalities (horizontal nystagmus, hypometric saccades, abduction deficits, esotropia, ptosis), mild to profound mental retardation with ventriculomegaly and/or generalised brain atrophy, poor or absent speech, and, in some, hyperreflexia and/or seizures [21].

Mutations in the mGluR1 gene cause cerebellar ataxia also in mice: A spontaneous mutation in the ligand-binding region of mGluR1 has been found to underlie ataxia in the recoil wobbler (rcw) strain of ataxic mouse [79]. Disruption of mGluR1 in mice by homologous recombination-mediated gene targeting was associated with atactic gait and intention tremor, although the gross anatomy of the cerebellum was widely normal, as was the excitatory synaptic transmission from PFs and CFs to PCs [46]. However, LTD (but not short-term synaptic plasticity) is clearly impaired in mGluR^−/−^ mice [46], and multiple (instead of single) innervation of CFs to PCs was observed [46, 80–82]. Similarly, no basic anatomical abnormalities were found in the hippocampus in mGluR1-deficient mice [45]; in contrast to the cerebellum, LTD was intact, but (mossy fibre) LTP and learning were impaired [45, 80, 83]. In rescue mice, all effects could be reversed in a dose-dependent manner by reconstitution of mGluR1 signalling [84].

Testing for spinocerebellar ataxia (SCA; types 1, 2, 3, 6, 7 and 17), Friedreich’s ataxia (FRDA), and fragile-X tremor-ataxia syndrome (FXTAS) has been carried out in one patient with mGluR1 antibodies and was negative [36].

## Anti-Homer-3

### Clinical, paraclinical and epidemiological features

The index patient (65/F) presented with vertigo, vomiting, dysarthria and severe subacute limb and gait ataxia. Ataxia was irreversible [85]. A second patient (38/M) also presented with nausea, vomiting and a pancerebellar syndrome but, in addition, developed signs of encephalitis including drowsiness, confusion and complex partial seizures. In this patient, elevated opening pressure and papilloedema was noted [86]. CSF analysis revealed lymphocytic pleocytosis in both cases (29 and 60 cells/μl); signs of intrathecal IgG synthesis were present in one patient. Brain MRI was normal at first examination in both patients, with no available follow-up in patient 1 and mild atrophy of the vermis and cerebellar hemispheres in patient 2 after 10 months. Onset of disease was subacute in both cases. Two additional (as yet unpublished) cases of ACA and Homer-3 antibodies have recently been diagnosed by us. No evidence has been found for a role of anti-Homer-3 in patients with chronic cerebellar ataxia (*n* = 27), patients with opsoclonus–myoclonus syndrome (*n* = 20) or healthy subjects (*n* = 20) [85, 86].

#### Association with tumours

Repeat tumour screening was negative in both published patients, with a follow-up period of 6 years in patient 1. One of the two as yet unpublished patients diagnosed at our laboratory had lung cancer (no data in the second), but no more detailed information is available.

#### Outcome and prognosis

While patient 1 did not respond to steroids, partial improvement was noted in patient 2 following treatment with IVIG and steroids. At last follow-up (72 and 24 months, respectively), patient 1 had severe ataxia, but patient 2 was still able to walk without help and carry out basic daily activities independently. It has been speculated that the suboptimal treatment response in patient 1 was due to the fact that significant Purkinje cell loss may occur very early in the clinical course, as seen in other antibody-mediated forms of ACA such as anti-Yo syndrome [86].

#### Antigen

Homer-3 is a constitutively expressed member of the Homer family of postsynaptic density (PSD) scaffolding proteins, which are characterised by enabled/vasodilator-stimulated phosphoprotein homology 1 (EVH1) domains. The EVH1 domain binds ligands on other proteins, including group I mGluRs, IP3 receptors, ryanodine receptors and Shank proteins. Homer-3 is thought to cross-link the cytoplasmic C-terminus of mGluR1 (especially the mGluR1a isoform [42, 43]) to ITPR1, both of which contain a proline-rich ‘Homer ligand’ (PPXXFR) [42]. Five isoforms of Homer-3 produced by alternative splicing are known to date in human. Besides Homer-3, two other Homer proteins with several isoforms have been described [42, 44, 87–90]. The various Homer proteins and isoforms are thought to modify differentially synaptic mGluR properties including mGluR1 clustering, mGluR1-ITPR linkage and, functionally, the capability of mGluRs to trigger calcium responses [42–44, 91, 92]. Ango et al. [41] suggested that Homer-3 prevents the so-called agonist (glutamate)-independent, constitutive activity specifically observed with isoform a of the mGluR1 receptor [93]. In the cerebellum, Homer-3 co-immunoprecipitates with structurally highly related Homer-1b [94], which influences translocation of the mGluRs from the ER to the plasma membrane, as well as with mGluR1 and ITPR1 [42]. Homer-3 may be regulated to some extent by the immediate-early gene product Homer-1a, its direct competitor on mGluR1a, which disrupts its binding to that receptor [41]. The coupling function of Homer-3 and thus the postsynaptic molecular architecture in response to synaptic activity in PCs has been proposed to be regulated by calcium/calmodulin-dependent protein kinase II (CaMKII)-mediated phosphorylation [95]. While non-phosphorylated Homer-3 is found mainly in the PSD, phosphorylated Homer-3 was found mainly in the cytosolic fraction [95]. Together with Shank, the Homer proteins form a mesh-like matrix structure that has been proposed to serve as a structural framework and as an assembly platform for other PSD proteins [96–99]. A coiled-coil domain near the C-terminus allows formation of multimeric structures within the Homer family, and tetramerised Homer proteins are assumed to be required for structural integrity of the dendritic spines and recruitment of proteins to synapses [96].

#### Immunohistochemistry

**Fig. 4:**
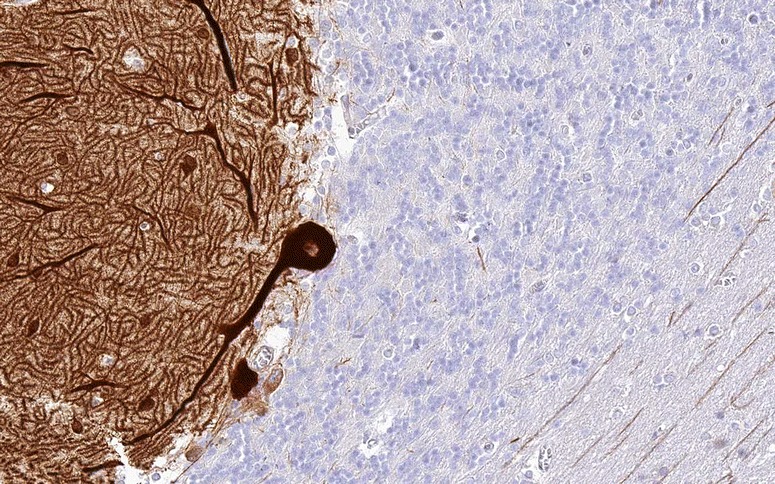
Expression of Homer-3 in the human cerebellum as demonstrated by IHC (modified image from the Human Protein Atlas image database [101]). Note that the main panel and the inset show different sectional planes

**Fig. 5:**
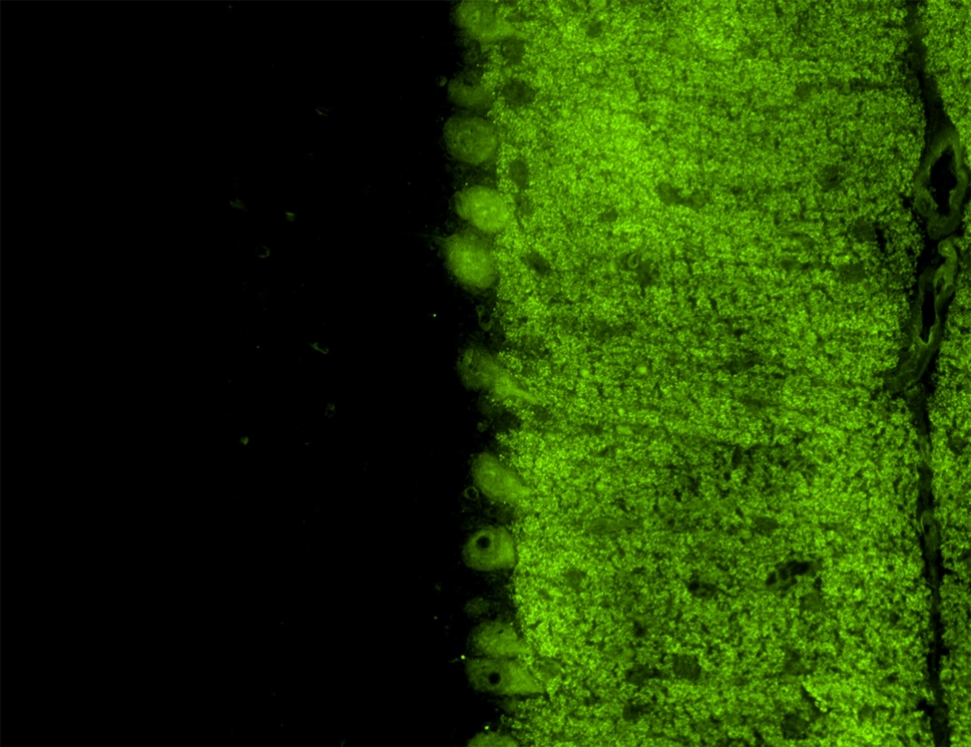
Binding of anti-Homer-3 antibody from a patient with ACA to a mouse cerebellum tissue section. The patient antibody was detected by use of a goat anti-human IgG secondary antibody labelled with Alexa Fluor®488 (green)

Homer-3 is expressed at high level in PCs, where it is enriched in the dendritic spines, more precisely in the PSD of the PC/PF synapses (Fig. 4). However, it is also present in the somata and has been found in PC axons [89]. At lower levels, Homer-3 is expressed also in the cortex and hippocampus. In the latter, it is predominantly localised in the CA2 and CA3 regions (in contrast to Homer 1 and 2, which are more strongly expressed in the CA1 and CA2 regions) [89]. Outside the CNS, Homer-3 has been detected in thymus and lung. Using conventional biotin-avidin IHC on paraformaldehyde-fixed rat and human cerebellum tissue sections, binding of IgG from anti-Homer-3-positive sera to the molecular layer and, less intensely, the PC cytoplasm (but no other brain regions) has been found [85, 86]. Anti-Homer-3 autoantibodies are also detectable by IIF on unfixed or formalin-fixed frozen sections of mouse, rat or primate cerebellum tissue (Fig. 5).

#### Antigen-specific assays

Currently, a CBA employing HEK293 transfected with human Homer-3 (Euroimmun), a mixed phage display assay [85], Western blot assays using cerebellum sections from primate [74], rat, wild-type (47 kDa) and Homer-3-deficient mice [85] and an immunoblot assay employing a glutathione S-transferase-tagged Homer-3 [86] are available for use in scientific studies. A competitive inhibition IHC assay has been used to distinguish Homer-3 from mGluR1 [85].

#### CSF testing

CSF was not analysed for anti-Homer-3 in the only two patients whose cases have been published thus far.

#### Association with other autoantibodies

Anti-Homer-3 autoantibodies were not found in 32 sera from patients with other antibody-associated CNS disorders (14× anti-Tr, 17× anti-GAD, 1× anti-mGluR1). Conversely, anti-Homer-3-positive patients were reported to be negative for anti-Hu, -Yo, -Ri, -Ma1/2, -CV2/CRMP5, -Tr, -GAD, -amphiphysin and/or -NMDAR, -AMPAR, -GABABR, -mGluR1, -mGluR5, -LGI1 and -CASPR2.

#### Pathogenetic relevance

While the intracellular location of Homer-3 renders antibody-dependent cell- or complement-mediated cytotoxicity unlikely, the broad spectrum of functions and interactions of Homer-3 within PCs makes a functional impact of incorporated anti-Homer-3 IgG at least conceivable. Among other effects, blocking the interaction of Homer-3 with mGluR1a in PC could increase constitutive mGluR1 activity, as indicated by spontaneous inositol phosphate formation and spontaneous activity of calcium-dependent big K+ channel activity following Homer-3 knock-down in a cell culture model [41]. Passive transfer experiments that alone could prove a pathogenic effect of anti-Homer-3 are lacking so far.

#### Molecular genetics

To date, no mutations in the HOMER3 gene have been described in patients with SCA or other diseases.

## Anti-Sj/ITPR1

### Clinical, paraclinical and epidemiological features

Anti-ITPR1 (also termed anti-Sj) autoantibodies were first identified in 2010 and reported in 2014 [100]. So far, only four patients with anti-Sj/ITPR1 have been published, all of whom had cerebellar ataxia; however, we have identified another eight (as yet unpublished) cases in the meantime. Detailed clinical data are available only from a single case, a 28-year-old woman with a 10-year history of progressive ataxia of the upper limbs, dysarthria and gaze disturbances. MRI showed moderate cerebellar atrophy.

#### Association with tumours

The only patient with available data tested positive for a BRCA1 (breast cancer 1, early onset) gene mutation, which is associated with increased risk of cancer, but extensive tumour screening was negative. ITPR1 expression has been observed in breast cancer, liver cancer, lung cancer, melanoma, and lymphoma tissue by IHC as well as in a number of tumour cell lines [101].

#### Outcome and prognosis

The disease did not respond to steroids and ten cycles of PEX, but progression spontaneously came to a halt 3 years later. At last follow-up (9 years after onset), the patient was still able to work full-time in an office.

#### Antigen

**Fig. 6:**
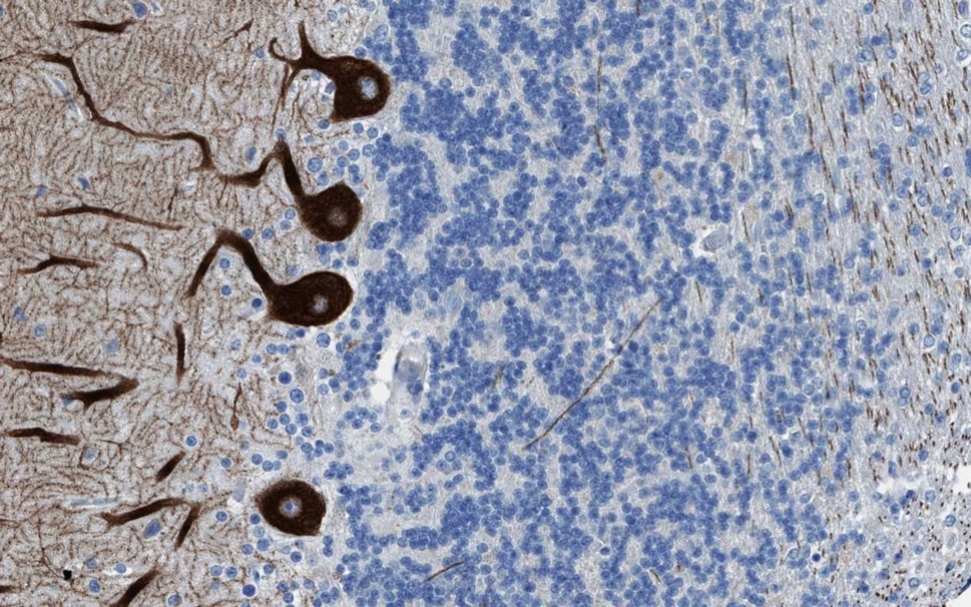
Expression of ITPR1 in the human cerebellum as demonstrated by IHC using an affinity-isolated rabbit antibody to human ITPR1 (Atlas antibodies, HPA016487). Modified image from the Human Protein Atlas image database [101]

ITPR1 (also termed IP3RI) is a ligand-gated non-selective cationic channel with sequence and functional homology with the ryanodine receptor. It is specifically gated by inositol 1,4,5-trisphosphate (IP3), a second messenger produced by phospholipase C through a G protein-dependent mechanism. ITPR1 is enriched in PCs (Fig. 6) [102–104], where it is expressed mainly in the smooth ER membrane (and to a lesser degree on rough ER and nuclear envelope). Being involved in postsynaptic calcium responses by triggering Ca^2+^ release from the smooth ER as the main intracellular Ca^2+^ store following stimulation of mGluR1, to which it is physically coupled by Homer-3, ITPR1 plays an essential role in PC function.

To date, eight isoforms of ITPR1 produced by alternative splicing have been described [105]. Besides its IP3-binding domain, which is located near the N-terminus, ITPR1 contains a transmembrane spanning domain near the C-terminus, a coupling domain in the middle of the molecule, at least two consensus protein kinase A phosphorylation sites and at least one consensus ATP-binding site [105]. Among the many proteins suggested to interact with ITPR1, CARP VIII and IP_3_R-binding protein released with IP_3_ (IRBIT) regulate its IP3 sensitivity [2, 106].

Of note, ITPR1-mediated release of Ca^2+^ from the ER also plays an important role in the induction of apoptosis [107–109]. Accordingly, inhibition or loss of inositol trisphosphate receptors [110–112] as well as mutation in the N-terminal suppressor/coupling domain of ITPR1 have been shown to suppress apoptosis [113].

#### Immunohistochemistry

**Fig. 7:**
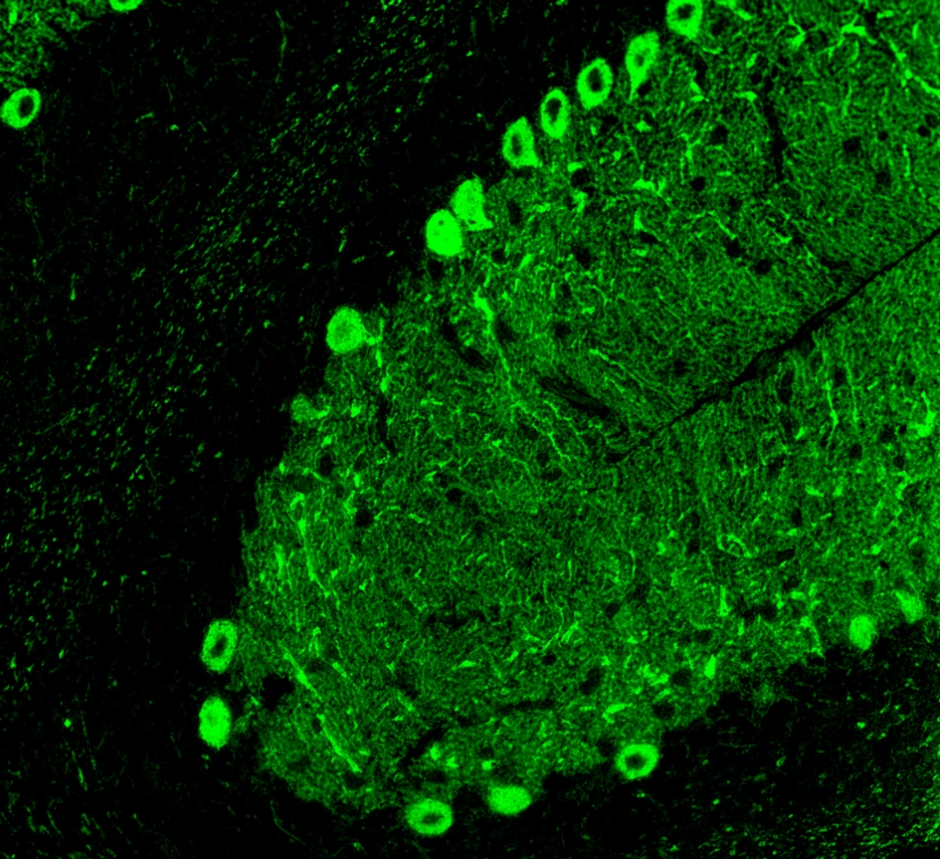
Binding of IgG from a patient with ITPR1-Ab-positive ACA as determined in a recombinant cell-based assay to formalin-fixed rat cerebellum tissue. Human IgG was detected using a goat anti-human IgG secondary antibody labelled with Alexa Fluor®488 (green fluorescence)

When tested by indirect immunofluorescence using snap-frozen cerebellum sections, anti-Sj/ITPR1 antibodies selectively bound to the entire dendritic tree in the cerebellar molecular layer including the dendritic spines [100, 102, 103] (Fig. 7), to the PC somata in the cerebellar PC layer, to the PC axons in the granular layer and the white matter and to the axonal terminals in the deep cerebellar nuclei. By contrast, granular cells, interneurons in the molecular layer and in the granular layer, the Bergman glial cells in the PC layer and the astrocytic and oligodendrocytic glial cells of the granular layer are spared. Anti-Sj/ITPR1 causes markedly stronger staining of the PC somata (somata ≥ dendrites) than anti-Ca/ARHGAP26 (somata < dendrites). In our experience, mouse and rat tissues seem to be more sensitive than primate tissue.

ITPR1 has also been detected in neurons in the CA1 region of the hippocampus, in the caudate nucleus and putamen, in the cerebral cortex [114], in the presynaptic terminals of photoreceptor and bipolar cells [115] and in the plasma membrane of the olfactory cilia [116–118]. High-titre samples may bind to smooth muscles on enteric tissue sections but spare the plexus myentericus.

Double-labelling experiments with a commercial antibody to ITPR1 can be employed to verify the presence of anti-Sj/ITPR1 antibodies when suspected (see [100] for an example).

#### Antigen-specific assays

A CBA employing HEK293 cells transfected with recombinant mouse ITPR1 and a dot–blot assay employing rat ITPR1 purified from brain tissue are available at the authors’ institution [100]. Specific neutralisation of the IHC reaction by preadsorption of patient sera with purified native rat ITPR1 has been used to confirm the presence of anti-ITPR1 [100].

#### CSF testing

Anti-Sj/ITPR1 has been identified from serum samples. Whether ITPR1 enters the CNS exclusively from the periphery or is also produced intrathecally is currently unknown. So far, testing of serum samples is recommended. However, given that many autoantibodies in neurological disease are produced intrathecally [119–122] and that some are occasionally detectable only in the CSF [123], testing of CSF samples could potentially be useful in serum-negative cases.

#### Association with other antibodies

In the patients reported thus far, no association has been found with anti-Ca/ARHGAP26, anti-Hu, anti-Ri, anti-Yo, anti-Ma, anti-Ta, anti-CV2/CRMP5, anti-amphiphysin, ANNA-3, PCA-2, or anti-Tr/DNER, anti-Homer-3, anti-mGluR1, anti-CARP VIII, anti-AQP4, anti-myelin oligodendrocyte glycoprotein, anti-NMDA receptor, anti-AMPA receptors 1 and 2, anti-GABABR, anti-dipeptidyl-peptidase 6 (DPPX), anti-LGI1, anti-CASPR2, anti-PKCγ, anti-Zic4, anti-GAD, anti-amphiphysin or anti-GluRδ2 [100].

#### Pathogenetic relevance

As passive transfer experiments using IgG from anti-ITPR1-positive patients have not yet been performed, no direct evidence for a pathogenic impact of the antibody is currently available. Indirect evidence suggesting a potential pathogenic role of anti-ITPR1 include its high specificity for PCs and the association of ITPR1 defects with SCA, together with the fact that it mainly belongs to the IgG1 subclass [100] and is usually present at high titres [100]. On the other hand, ITPR1 is primarily an intracellular antigen and may not be accessible to antibodies in vivo. It is therefore possible that the antibody has diagnostic but no pathogenic impact, similar to the situation in many paraneoplastic neurological syndromes. However, surface localisation has also been reported under certain circumstances [124–128], warranting further investigation.

#### Molecular genetics

Mutations in the ITPR1 gene have been implicated in both SCA15 and SCA29. SCA15, which has to be shown to be identical to SCA16 [129], is an autosomal dominant, very slowly progressive form of cerebellar ataxia with adult onset. In addition to ataxia, action and postural tremor, pyramidal tract and dorsal column involvement and gaze palsy have been noted. MRI revealed cerebellar atrophy, predominantly affecting the vermis [130]. In most affected families, large exon deletions have been found to underlie the disorder [25, 129–131]. Accordingly, diagnosis is based on gene dosage studies rather than direct gene sequencing in such cases [25]. In Japanese patients, deletions involving the entire ITPR1 gene [132] and a heterozygous 8581C-T transition in exon 25 of the ITPR1 gene, resulting in a P1059L substitution in the ITPR1 gene [132], were identified.

SCA29 is an autosomal dominant disorder characterised by gait and limb ataxia with childhood onset and delayed motor and cognitive development. MRI shows cerebellar atrophy [133]. Two different heterozygous mutations in the ITPR1 gene, a 4657G-A transition resulting in a val1553-to-met (V1553M) substitution and a heterozygous 1804G-A transition in the ITPR1 gene resulting in an asn602-to-asp (N602D) substitution, respectively, have recently been found to underlie SCA29 in two affected families [133].

## Anti-CARP VIII

### Clinical, paraclinical and epidemiological features

So far, two patients with ACA and anti-CARP VIII have been reported. The index patient was a 77-year-old woman who presented with vertigo, severe limb and gait ataxia, dysarthria and horizontal nystagmus; symptoms developed within just 1 week [134]. In a second patient, a 69-year-old woman, intention tremor of the upper extremities, gait ataxia, cerebellar dysarthria and vertical nystagmus developed within 2 weeks; further symptoms included headache, vertigo and vomiting [135]. While brain MRI was normal in patient 1, repeat MRI showed progressive cerebellar atrophy 6 months later in patient 2. Lumbar puncture revealed a predominantly lymphocytic pleocytosis in both patients (60 and 290 cells/μl, respectively); CSF-restricted oligoclonal bands (OCB) were positive in the first patient and were not tested in the second patient.

#### Association with tumours

The index patient had been diagnosed with a nodular recurrence of malignant melanoma around 3 months before onset of cerebellar ataxia [134]. This is the first reported case of melanoma-associated paraneoplastic cerebellar degeneration (PCD). While the patient’s tumour was not examined for CARP VIII expression, the protein was shown to be expressed rarely in frozen sections of malignant melanomas from other patients [101, 134]. Anti-CARP VIII autoantibodies were not found in any of 52 patients with melanoma but no paraneoplastic syndromes [134]. The second patient was diagnosed with nodular recurrence of an ovarian papillary serous cystadenocarcinoma that had been resected and treated with chemotherapy 4 years prior to onset of ataxia. IHC of biopsy material from that patient revealed robust expression of CARP VIII in the tumour cells [135]. Besides in melanoma and ovarian carcinoma cells, expression of the normally neuron-restricted CARP VIII has also been found in colorectal and non-small-cell lung cancer cells [101].

#### Outcome and prognosis

The index patient developed a pancerebellar syndrome despite treatment with IVIG and died 1 year after onset of ataxia [134]. The clinical evolution was also unfavourable in the second patient, who became wheelchair-bound and developed severe dysarthria despite tumour removal and IVIG therapy [135].

#### Antigen

**Fig. 8:**
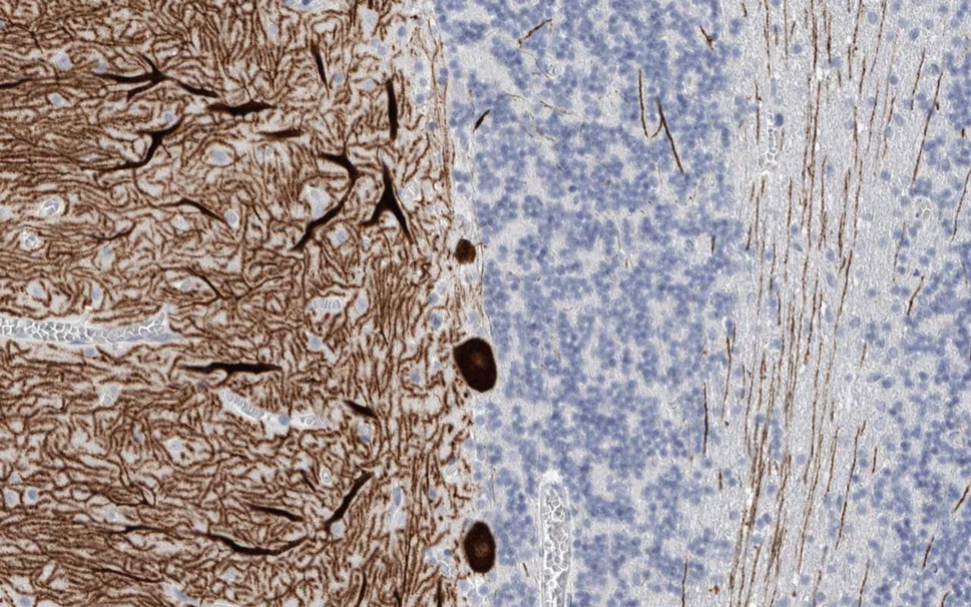
Expression of CARP VIII in the human cerebellum as demonstrated by IHC (modified image from the Human Protein Atlas image database [101])

CARP VIII (also termed carbonic anhydrase VIII; encoded by CA8) belongs to an 11-member family of zinc metalloenzymes. While it shows sequence identity to other members of the cerebellar ataxia gene family and has a central CA motif, it lacks CA activity due to the absence of zinc-binding histidine residues [136, 137]. CARP VIII has been shown to reduce the affinity of ITPR1 for IP3 by its binding to the modulatory domain (residues 1387 to 1647) of that receptor via its residues 45 to 291 [2]. CARP VIII is predominantly expressed in PCs (Fig. 8) [2, 101] and is believed to have an important function in the development and maturation of these cells [138, 139].

#### Immunohistochemistry

**Fig. 9:**
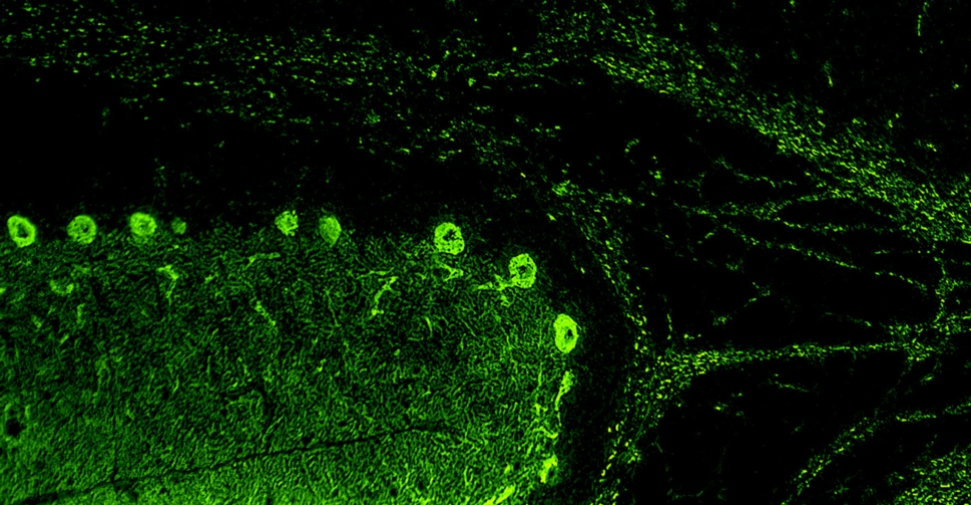
Binding of IgG from a patient with CARP VIII-Ab-positive ACA (as determined using a recombinant cell-based assay) to formalin-fixed rat cerebellum tissue. Human IgG was detected using a goat anti-human IgG secondary antibody labelled with fluorescein isothiocyanate (green fluorescence)

In PCs, CARP VIII colocalises with ITPR1 [2], resulting in a similar IHC pattern in the two diseases (Fig. 9). CARP VIII immunoreactivity is highest in PCs of the cerebellum [2], with lower levels of expression in other areas of the brain, including the olfactory bulb, the lateral nuclei of the thalamus and a few isolated small neurons throughout the cortex and hippocampus [134, 140, 141]. Anti-CARP VIII has been shown to stain intensely the cytoplasm of the PC somata, the PC dendritic tree and the PC axons both in rat and human cerebellum [134], as well as the synaptic terminals in the deep cerebellar nuclei [135]. Outside the CNS, CARP VIII has been found in the lung, liver, adrenal glands and, weakly, in the bronchial epithelial cells and some tubules in the kidney cortex [134, 140, 141]. Of note, CARP VIII antibodies were detectable by IHC using avidin-biotin immunoperoxidase staining of frozen sections of paraformaldehyde-fixed rat tissues or of sections of snap-frozen acetone-fixed human tissues; by contrast, paraffin fixation abolished the staining [134].

#### Antigen-specific assays

A CBA using HEK293 cells transfected with human CARP VIII (Euroimmun) is available at the authors’ institution for use in scientific studies. The antibody was first identified by screening a cerebellar complementary DNA (cDNA) expression library with the patient’s serum, subcloning, purification and sequencing of positive plaques. Filters with the purified phage plaques were subsequently used for detecting anti-CARP VIII in a second patient [135]. Fusion proteins produced by subcloned positive colonies were used for establishing a Western blot (WB) assay and an IHC preadsorption assay [134]. The protein was also shown to be reactive with a 29-kDa band in rat and human cerebellum extract [134, 135]. Finally, a competitive IHC inhibition assay using patient serum and subsequently biotinylated anti-CARP VIII IgG obtained from the index patient has been reported [135]; however, the latter type of assay requires, as a limitation, concordance in epitope specificity between patients.

#### CSF testing

In the index patient, anti-CARP VIII autoantibodies were present at high titres both in serum (1:160,000) and in the CSF (1:10,000). The high CSF levels indicated possible intrathecal synthesis [134]. CSF was not analysed for CARP VIII in the second patient.

#### Association with other autoantibodies

In the two patients reported thus far, no concomitant anti-Hu, -Yo, -Ri, -Ma1, -Ma2, -CV2/CRMP5, -sex-determining region Y-box (SOX) 1, -Tr, -glutamic acid decarboxylase, -amphiphysin, -VGCC, -LGI1, -CASPR2, -NMDAR, -AMPAR, -GABABR, -DPPX, -mGluR1, -mGluR5, or -glycine receptor antibodies were detected.

#### Pathogenetic relevance

Although anti-CARP VIII autoantibodies were present at extremely high titres in patient 1 and were shown to belong to the IgG1 subclass in patient 2, a direct pathogenic role of the antibody is unlikely given the intracellular location of CARP VIII. However, results from passive transfer experiments are lacking thus far.

#### Molecular genetics

Mutations in the CA8 gene have been found to be associated with congenital cerebellar ataxia and mild mental retardation with or without quadrupedal locomotion 3 [142, 143]. In mice, a deletion in exon 8 of the Car8 gene has been found in both the autosomal recessive ataxic and dystonic ‘waddles’ (wdl) mouse [144] and the autosomal recessive ataxic ‘Rigoletto’ (rig) mutant mouse [145]. Mutant mice show a largely diminished spontaneous PF/PC excitatory transmission with fewer functional synapses, PC spines not forming synapses and abnormal PC spines contacting multiple synaptic varicosities [145]. Absence of CARP VIII messenger RNA (mRNA) has been noted also in the atactic ‘lurcher’ mutant mouse [146]. In wdl mice, Car8 mutations did not influence ITPR1 expression [144].

## Note to the reader

In Part 2 of this series, we will review the current knowledge on anti-PKCγ-, anti-GluRδ2-, anti-Ca/ARHGAP26- and anti-VGCC antibody-associated ACA [212]. In Part 3, we will focus on anti-Tr/DNER-,anti-Nb/AP3B2-, anti-Yo/CDR2- and PCA-2-associated ACA, discuss diagnostic pitfalls and provide a summary and outlook [213].

## Abbreviations

Aa: amino acids
ACA: autoimmune cerebellar ataxia
AChR: acetylcholine receptor
ADEM: acute disseminated encephalomyelitis
AF: Alexa Fluor®
AGNA: anti-glial cell nuclear antibodies
AMPA: α-amino-3-hydroxy-5-methyl-4-isoxazolepropionic acid
ANA: anti-nuclear antibodies
ANNA: anti-neuronal nuclear antibody
APTX: aprataxin
AQP4: aquaporin-4
ARHGAP26: Rho GTPase-activating protein 26
ATXN1: ataxin-1
BAR: bin–amphiphysin–Rvs
beta-NAP: neuronal adaptin-like protein
CARP VIII: carbonic anhydrase-related protein VIII
CASPR2: contactin-associated protein-2
CBA: cell-based assay
cDNA: complementary DNA
CDR2: cerebellar degeneration-related protein 2
CDR2L: CDR2-like
CDR3: cerebellar degeneration-related autoantigen-3
CF: climbing fibre
CHO: Chinese hamster ovary
CNS: central nervous system
CSF: cerebrospinal fluid
CTL: cytotoxic T lymphocytes
DAG: diacylglycerol
DAPI: 4',6-diamidino-2-phenylindole
DNA: deoxyribonucleic acid
DNER: delta notch-like epidermal growth factor-related receptor
DPPX: dipeptidyl-peptidase 6
EA2: episodic ataxia type 2
EGF: epidermal growth factor
ELISA: enzyme-linked immunosorbent assay
ER: endoplasmic reticulum
FHM1: familial hemiplegic migraine 1
FITC: fluorescein isothiocyanate
GABA: γ-aminobutyric acid
GABABR: GABA type B receptor
GAD: glutamic acid decarboxylase
GAP: Rho GTPase-activating protein
GFAP: glial fibrillary acidic protein
GL: granular layer
GluR: ionotropic glutamate receptors
GluRδ2: glutamate receptor delta 2
GRAF1: GTPase regulator associated with focal adhesion kinase 1
HEK: human embryonic kidney
IB: immunoblot assay
ICC: immunocytochemistry
IIF: indirect immunofluorescence
IgA: immunoglobulin A
IgG: immunoglobulin G
IgM: immunoglobulin M
IHC: immunohistochemistry
IP: immunoprecipitation assay
IP3: inositol 1,4,5-trisphosphate
IRBIT: IP_3_ receptor-binding protein released with IP3
ITPR1: inositol 1,4,5-trisphosphate receptor, type 1
IVIG: intravenous immunoglobulins
IVMP: intravenous methylprednisolone
kDa: kilodalton
LEMS: Lambert–Eaton syndrome
LGI1: leucine-rich, glioma inactivated 1
LTD: long-term depression
LTP: long-term potentiation
mGluR1: metabotropic glutamate receptor 1
ML: molecular layer
MRI: magnetic resonance imaging
mRNA: messenger RNA
NMDA: *N*-methyl *D*-aspartate
NMO: neuromyelitis optica
NSCLC: non-small cell lung cancer
NSE: neuron-specific enolase
OCB: oligoclonal bands
PC: Purkinje cell
PCA: Purkinje cell antibody
PCD: paraneoplastic cerebellar degeneration
PCL: PC layer
PF: parallel fibre
PIP_2_: phosphatidyl 4,5-bisphosphate
PKCγ: protein kinase C gamma
PLCβ3: phospholipase Cβ3
PSD: postsynaptic density
RACK: receptors for activated C kinases
RhoA: ras homolog gene family, member A
RIA: radioimmunoprecipitation assay
RNA: ribonucleic acid
SCA: spinocerebellar ataxia
SCLC: small cell lung cancer
SOX: sex-determining region Y-box
TRPC: canonical transient receptor potential channel
TUNEL: TdT-mediated dUTP-biotin nick end labelling
VGCC: voltage-gated potassium channel
VGKC: voltage-gated potassium channel
WB: Western blot
WM: white matter
